# An Evaluation of the Healthiness of the Indian Packaged Food and Beverage Supply

**DOI:** 10.3390/nu9101103

**Published:** 2017-10-09

**Authors:** Alexandra Jones, Elizabeth Dunford, Rachel Crossley, Sudhir Raj Thout, Mike Rayner, Bruce Neal

**Affiliations:** 1 Food Policy Division, The George Institute for Global Health, UNSW Sydney, Sydney 2042, Australia; edunford@georgeinstitute.org.au (E.D.); bneal@georgeinstitute.org.au (B.N.); 2 Charles Perkins Centre, University of Sydney, Sydney 2006, Australia; 3 Carolina Population Center, University of North Carolina at Chapel Hill, Chapel Hill, NC 27516, USA; 4 Access to Nutrition Foundation, 3511 Utrecht, The Netherlands; rachel.crossley@accesstonutrition.org; 5 The George Institute for Global Health, Hyderabad 500034, India; traj@georgeinstitute.org.in; 6 Nuffield Department of Population Health, University of Oxford, Oxford OX37BN, UK; mike.rayner@dph.ox.ac.uk; 7 Division of Epidemiology and Biostatistics, Imperial College London, London SW7 2AZ, UK

**Keywords:** nutrient profiling, packaged foods, public health nutrition, India, food manufacturers

## Abstract

Availability of less-healthy packaged food and beverage products has been implicated as an important driver of obesity and diet-related disease. An increasing number of packaged foods and beverages are sold in India. Our objective was to evaluate the healthiness of packaged foods sold by India’s largest manufacturers. Healthiness was assessed using the Australian Health Star Rating (HSR) system and the World Health Organization’s European Regional Office (WHO Euro) Nutrient Profile Model. Sales-value-weighted mean healthiness and the proportions of “healthy” products (using a validated HSR cut-off of ≥3.5, and products meeting WHO Euro criteria as healthy enough to market to children) were calculated overall, by company and by food category. Nutrient information for 943 products sold by the 11 largest Indian manufacturers was obtained from nutrient labels, company websites or directly from the manufacturer. Healthiness was low overall (mean HSR 1.8 out of 5.0 stars) with a low proportion defined as “healthy” by both HSR (17%) and also by WHO Euro criteria (8%). There were marked differences in the healthiness of similar products within food categories. Substantial variation between companies (minimum sales-value-weighted mean HSR 0.5 for Company G, versus maximum HSR 3.0 for Company F) was a result of differences in the types of products sold and the nutritional composition of individual products. There are clear opportunities for India’s largest food companies to improve both the nutritional quality of individual products and to improve their product mix to include a greater proportion of healthy products.

## 1. Introduction

Malnutrition in all its forms is a leading cause of death and disease globally [1]. While 800 million people still go hungry, there are now 1.9 billion adults and 42 million children who are overweight or obese [1]. Countries such as India now face a “double burden” of malnutrition: experiencing ongoing high rates of undernutrition, while simultaneously facing a rapid rise in overweight-, obesity- and diet-related non-communicable diseases (NCDs). India now ranks third after the US and China in the number of obese people in the population, and 22% of children and adolescents are classified as overweight or obese [2]. Together these conditions incur huge economic and health costs [1]. 

As in many low- and middle-income countries, India is experiencing a “nutrition transition”. National nutrition surveys from the past few decades show changing consumption patterns: a traditional diet based upon fruits, vegetables and unprocessed cereals, and legumes is being replaced by an increasing amount of highly processed and packaged products. This shift has been seen particularly among high- and middle-income groups [3]. Food and groceries currently account for around 31% of India’s consumption basket. While packaged foods are estimated to make up only 6% of household spending, the sector is expanding rapidly. Increasing urbanization, improving distribution networks, and growing disposable incomes are likely to drive consumers to buy more of these products in the future [4]. Unprecedented availability and aggressive marketing of these products—typically energy dense and high in harmful fats, sugar and salt—is a key driver of unhealthy diets globally [5], creating an urgent need to understand the nutritional quality of the Indian packaged food supply. 

Nutrient profiling provides a means of differentiating foods and drinks that are more likely to be part of a healthy diet from those that are less healthy. A number of nutrient profile models have been developed by academics, governments, non-governmental organizations and the food industry for applications such as regulation of marketing of foods to children, product labeling logos or symbols, and the making of health and nutrient content claims. Nutrient profiling is a tool to quantify aspects of individual foods, and it is recognized by the World Health Organization (WHO) as a helpful method to use in conjunction with interventions aimed at improving the overall nutritional quality of diets [6]. 

Our objective was to use nutrient profiling to examine the healthiness of products made available by the largest food and beverage manufacturers in India. By comparing the healthiness of company product profiles overall, and differences between similar products, our goal was to make recommendations for how manufacturers and the Indian Government could act to promote healthier diets. 

## 2. Materials and Methods

**Figure 1:**
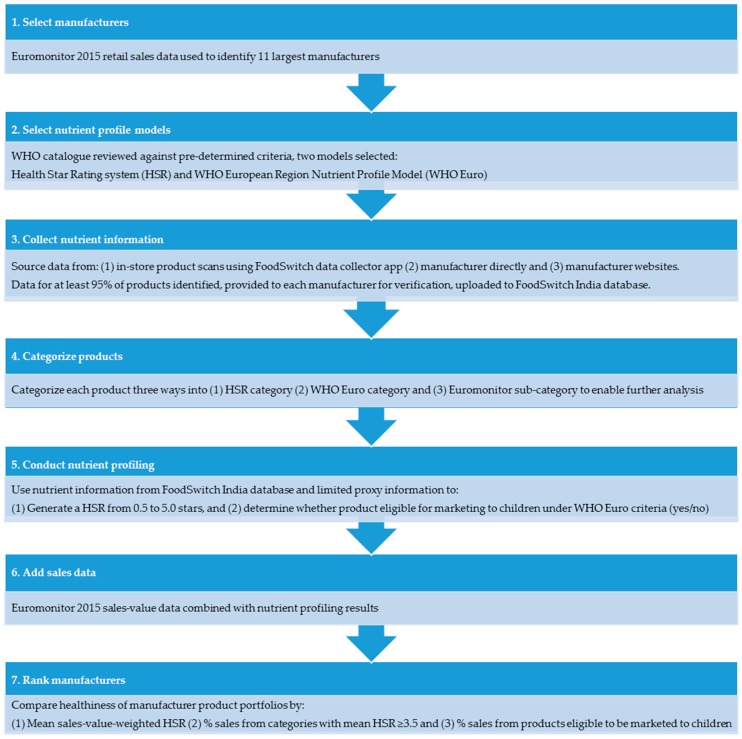
Methods and data sources.

This was a cross-sectional examination of packaged foods and beverages offered by the 11 largest food manufacturers operating in India by 2015 sales value (in alphabetical order by full name): Britannia Industries; Coca-Cola India Pvt. Ltd.; Gujarat Coop Milk Marketing Federation; Hindustan Unilever; ITC Limited; Karnataka Milk Federation; Mondelez International; Mother Dairy; Nestlé India; Parle Products Pvt. Ltd.; PepsiCo India, Inc. The project took place between March and November 2016 as a collaboration between The George Institute for Global Health and the Access to Nutrition Foundation [7]. Methods and data sources are summarized in Figure 1.

### 2.1. Nutrient Profile Models

There is no international consensus about the superiority of one particular nutrient profile model, in part due to the different purposes and contexts in which each model has been developed. We therefore started from the position that at least two models be used to assess products, to improve the validity of our findings. We reviewed 67 nutrient profile models catalogued by WHO [8] and by an iterative process of screening and review selected the two models that best met the following criteria; (1) developed with appropriate stakeholder consultation; (2) covered the majority of categories of foods and beverages; (3) designed to assess foods in the general market (i.e., not just school or hospital foods, for example); (4) well-validated with results published in peer-reviewed literature demonstrating that the model produces internally consistent classification of “healthy” and “unhealthy” foods, consistent with general nutrition principles; (5) enabled differentiation of nutritional quality within and between categories and (6) the algorithm was available in the public domain. 

The Australian HSR system is a front-of-pack interpretive nutrition labeling system designed to assist consumers to make healthier choices. The underlying nutrient profile model uses an algorithm that subtracts points for risk nutrients (overall energy, sodium, total sugar, saturated fat) and awards points for positive nutrients (fruit and vegetable content, protein, fiber and in some cases, calcium) to score products on the basis of their overall nutritional composition per 100 g or 100 mL for six broad categories [9,10]. Scores are then converted to a “Health Star Rating” from ½ to 5 stars in half star increments. Development was led by the Australian government in collaboration with industry, public health and consumer groups. The system has been implemented on Australian packaged foods since June 2014 on a voluntary basis [11].

The WHO Euro system is a nutrient profile model for use and adaptation by Member States of the WHO European Region when developing policies to restrict the marketing of unhealthy food and beverages to children [12]. Launched in 2015, the model operates by first requiring foods to be allocated to one of 20 categories. Products are then checked against category-specific compositional thresholds for nutrients and other food components. A product must not exceed any of the relevant thresholds for that product category on a per 100 g/mL basis to be considered healthy enough to be marketed to children. Results were dichotomized as “eligible for marketing” if they did not exceed the category thresholds specified in the model, or “marketing not permitted” if they did. In the absence of relevant Indian legislation in this area, the model was selected as a reasonable basis by which to determine products’ suitability to be marketed to children. 

### 2.2. Food Product Data

We sought data on all packaged foods and non-alcoholic beverages made by the 11 manufacturers. Nutrient information (energy, protein, sodium, carbohydrate, total sugar, total fat, saturated fat, and calcium per 100 g) was obtained either from product packaging, directly from the manufacturer or from company websites. Data were supplemented by in-store product scans undertaken between June and August 2016 at seven retail and wholesale outlets in Hyderabad, Bangalore, Delhi and surrounding areas. The period and geographic scope of collection was determined by the need to expand data collection until at least 95% coverage of each manufacturer’s product list (obtained directly or through company websites) was reached. Permission to photograph products was obtained from store owners. Data collectors attended each site and used a smartphone app to systematically photograph publicly-available nutrition information on product packaging. Data from all sources was uploaded to the FoodSwitch India database [10]. Where a product was captured more than once, information was extracted from the most recent photograph. Products collected in-store were checked against product portfolio information provided by food companies, and that available on company websites as well as those of two large Indian online retailers. At the end of the collection period in July 2016, companies were asked to review their data and offered an opportunity to make corrections or additions. Sales value data were obtained under license from Euromonitor International for each company for the 2015 period [13].

### 2.3. Product Definition and Categorisation

A product was defined as a unique item based upon the brand name and product description. Products sold in different serve size or packaging were not considered different products (i.e., a specific brand of cola sold in a 330 mL can and a 600 mL bottle were treated as a single product). All products were categorized according to the system developed by the Global Food Monitoring Group [14] with assignments stored in the FoodSwitch India database. This hierarchical system was designed to support monitoring of the nutrient composition of processed foods around the world.

FoodSwitch categories were mapped to the six HSR categories, to the 21 WHO Euro categories, and to the 50 Euromonitor International food and beverage sub-categories (Supplementary Table S1). Results were examined by subsets of the Euromonitor categories that included comparable products. Products exempt from displaying a nutrition label under Indian regulations were excluded (plain tea and coffee; condiments and herbs; unprocessed meat, poultry and fish) as were infant formula and baby foods because they are not consumed by the general population and the selected nutrient profile models were not appropriate for their evaluation.

### 2.4. Imputation of Missing Nutrition Data

Imputation was used to generate missing data required for application of the nutrient profile models (Supplementary Table S2). Proxy values for total fat, saturated fat, total sugar, sodium, fiber and “fruit vegetable nut and legume” (FVNL) content were derived from the full FoodSwitch India database using methods described previously [10]. In brief, the average nutrient value of the products with available data was calculated for each category and assigned to products in that category with missing data. For added sugars a standard proportion of total sugars was assumed and was specified at the category level. Specifically, for cakes and desserts, confectionery, sauces and beverages (excluding milk), total sugar values were assigned as added sugar. For milks and yoghurts, an amount of sugar of up to 6 g/100 g and 8 g/100 g respectively was considered to be naturally occurring based upon known concentrations of lactose in these products and any amount over this was assigned as added sugar. 

### 2.5. De-Identification of Companies

At the request of the journal editors and their legal advisors, we de-identified companies in our published results. Interested readers should refer to the related Access to Nutrition Foundation report [7] or contact the corresponding author directly to obtain the results in full without redactions.

## 3. Analysis

Product numbers, category and healthiness of products were summarized overall, by company, separately for foods and beverages, and by Euromonitor sub-category (where five or more products existed for two or more companies) using means, ranges and proportions as applicable. Comparisons were made based on mean HSR, proportions of products receiving an HSR ≥ 3.5 and proportions eligible to be marketed to children under WHO Euro criteria. The HSR for each product was calculated in accordance with the HSR Guidance for Industry established by the Australian government [9]. Nutrient profiling was done for products that had label data for energy content and at least two of following four key nutrients required to generate a HSR score: saturated fat, sugar, sodium or protein. Provided this minimum threshold of nutrient information was available on the product label, proxy values were applied for saturated fat, total sugar, fiber and sodium as necessary to run the HSR calculator. Plain water (whether still or carbonated) was assigned a HSR of 5.0 consistent with the HSR guidelines. The sales-value-weighted mean HSR for each company was calculated by multiplying the mean HSR of each sub-category for each company by the sales value for the relevant Euromonitor sub-category, summing the values obtained for the company and then dividing this figure by the total value of the company’s sales. Where a company did not command 0.1% or more market share in a Euromonitor sub-category, no sales data were available and products were excluded from the respective analyses.

The proportion of products in each company’s portfolio that could be classified as “healthy” was also used for ranking purposes. In the first case this was done by weighting the proportion of products with an HSR ≥ 3.5 using a similar method to that employed for obtaining sales-value-weighted mean HSR. This cut-off point was based on previous research indicating that healthy core foods with a HSR of ≥ 3.5 can be confidently promoted in public settings [15]. In the second case the proportion of products that met the WHO Euro criteria for marketing to children was used. To calculate WHO Euro eligibility, proxy values were used for total fat, saturated fat, sugar and sodium but only if the product was not missing three or more nutrients. Eligibility was determined category-by-category as per the model, which uses different nutrient criteria for each. All analysis was undertaken using STATA version 14.1.3 (StataCorp LP, College Station, TX, USA).

## 4. Results

### 4.1. The Comparative Healthiness of Company Product Portfolios

There were 1450 products identified and entered into the FoodSwitch India database. Of these, 59 were in excluded categories, 60 had insufficient data to enable any nutrient profiling, and 388 were duplicates. This left 943 unique products for analysis from the 11 companies across 37 Euromonitor sub-categories (Supplementary Table S3). Five out of the 11 companies (Company A, Company B, Company D, Company G and Company I) reviewed the data provided to them for checking, and made additions or amendments as necessary. 

Applying rules on proxy data a HSR was able to be determined for 918 of the 943 products, and eligibility for marketing to children under the WHO Euro system for 937.

**Figure 2:**
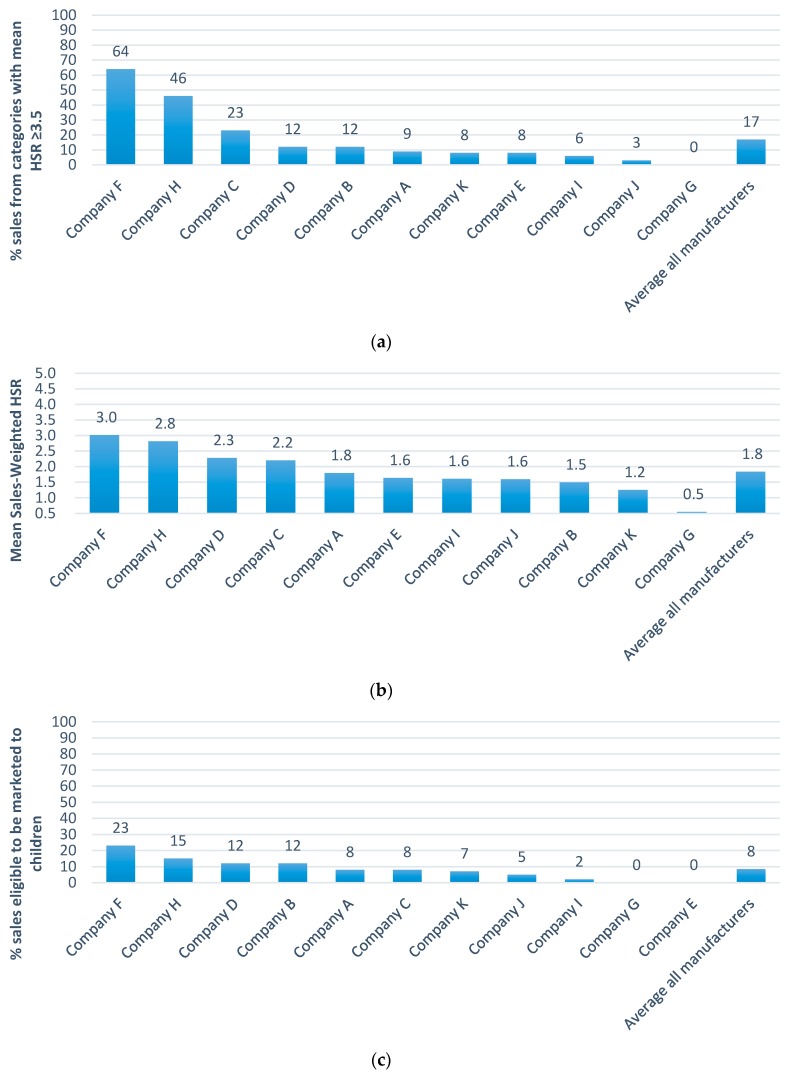
Ranking of companies by (**a**) mean sales-weighted Health Star Rating (HSR); (**b**) proportion of healthy product sales (HSR ≥ 3.5) and (**c**) proportion of product sales eligible for marketing to children.

The mean sales-value-weighted HSR for all products was 1.8. Company F was the company with healthiest portfolio (mean HSR = 3.0) and Company G the company with the least healthy (mean HSR = 0.5) (Figure 2). The high ranking for Company F was attributable to the large proportion of the value of its sales deriving from drinking milks and yoghurts and the low value for Company G being a consequence of the many confectionery items in its portfolio. 

Only 17% of all products achieved a HSR of ≥3.5 (Figure 2) and 54% had a HSR of 1.5 stars or below (Supplementary Table S4). Company F had the highest sales-value-weighted proportion of products receiving 3.5 stars or more (64% of sales), followed by Company H (46%) and Company C (23%). Company J (3%) and Company G (0%) had the lowest proportions because both predominantly make confectionery and biscuits (Supplementary Table S3). Likewise, a very low proportion of products (based on sales) were eligible to be marketed to children under the WHO Euro scheme (8%) (Figure 2). Company F again had the largest proportion of products eligible for marketing to children (23%). Eligible products were a mix of healthier dairy options, soups and some cereals (oats). Confectionery, cakes and sweet biscuits are ineligible for marketing to children under the WHO Euro nutrient profile model regardless of nutrient content, affecting results for companies that make a large number of these products.

### 4.2. The Comparative Healthiness of Different Product Types

**Figure 3:**
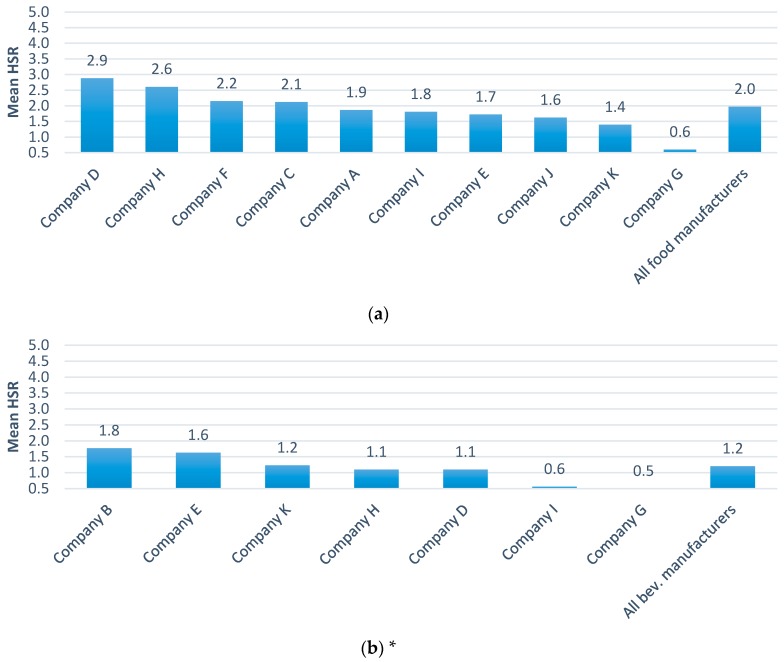
Ranking of companies by mean HSR a) foods and b) beverages. (**a**) Foods (10 companies); (**b**) Beverages (7 companies). * Euromonitor categorization includes dairy drinks as foods, so results for milks and drinking yoghurts appear in foods. Manufacturers only included if they make more than five foods or beverages.

**Figure 4:**
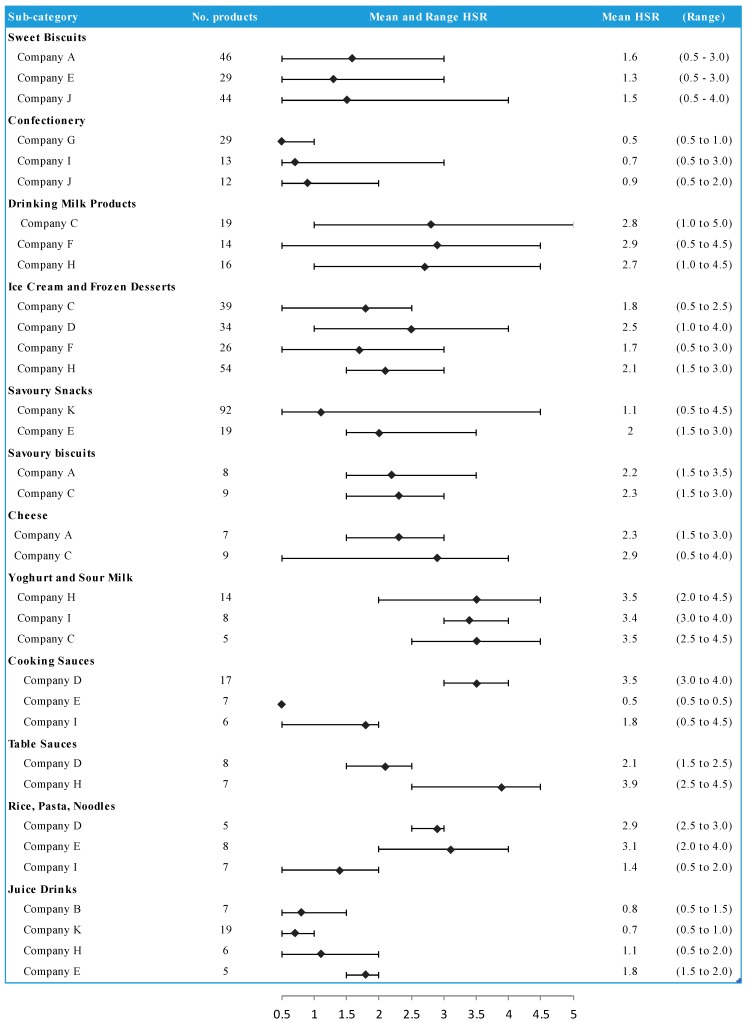
Figure 3 Mean and range HSR by company for selected Euromonitor subsets.

Foods were on average healthier than beverages (mean HSR 2.0 versus HSR 1.2) (Figure 3). Company B ranked first in mean healthiness of beverages based upon sales of plain bottled water and plain soda water mixers which receive an HSR of 5.0 but most other beverages had HSR values of 2.0 or less. The mean and range HSRs for Euromonitor sub-categories were compared where two or more companies sold five or more products (Figure 4, Supplementary Table S5). These analyses showed substantial differences between companies for categories such as rice, pasta and noodles, with yoghurts and sour milks showing more similar results across companies. Within most Euromonitor subcategories there was large variation in the HSRs of similar products (e.g., drinking milks ranged from 0.5 to 4.5) suggesting opportunities for reformulation (Supplementary Table S5).

## 5. Discussion

The overall mean healthiness of products from India’s 11 largest food and beverage companies was low and the mean healthiness of product portfolios varied substantially between companies. Differences in healthiness between companies reflect primarily differences in the types of products offered in their portfolios. Higher-ranking companies tended to produce mainly dairy products and lower-ranking companies mainly confectionery and savory snacks. To a lesser extent the variation also reflects differences in the healthiness of products within the same categories. For example, Company H, Company F and Company C all produce a variety of dairy products but mean healthiness based on sales for Company C was lower than other companies. 

The rankings of companies from healthiest to least healthy varied depending upon whether the comparison was based upon mean HSR, the proportion of products defined as “healthy” by a HSR cut point of ≥3.5 or eligibility for marketing to children under the WHO Euro criteria, but were broadly comparable across the three indicators. This is reassuring, though not unsurprising, given that all measures are based upon nutrient profile models. Healthiness defined by mean HSR generally resulted in greater variation between companies and this likely reflects the more detailed information captured by the continuous nature of the HSR assignment. This continuous measure of the HSR also had the advantage of providing a measure of spread of the nutritional quality of evaluated products, which is not provided by the simple dichotomization of portfolios into healthy or not. The assessments made according to eligibility for marketing to children did, however, provide a stark and very easily understood message about the poor nutritional quality of Indian packaged foods. A number of companies had no products currently healthy enough to be eligible for marketing to children, suggesting the importance of the Indian Government developing legislation in this area (Box 1). Since this analysis was completed, the WHO South-East Asia Region has finalized an adapted version of the WHO Euro criteria that may provide an appropriate basis to develop regulation [16].

The within-category comparisons of similar foods and beverages showed often marked differences in the healthiness of similar products. This was particularly the case for foods, as beverages tended to be more nutritionally comparable. This variation highlights the potential for manufacturers to reformulate products to healthier compositions (Box 1). There were also categories where one company had a range of products that were clearly healthier than those of a competitor. Companies could address this problem by adopting well-established, verified nutrient profiling models to underpin reformulation and new product development.

The analyses benefitted from sales-value-weighting which, while only able to be done at the sub- category (not individual product) level, provided an estimate of the real impact that each company is likely to be having on the healthiness of Indian diets. Rankings done using unweighted averages of the healthiness of product portfolios were similar to the primary findings of the project but could be unduly influenced by products that achieve only small sales volumes (Supplementary Figure S1). The validity of the sales-value-weighting process would be enhanced by the availability of product-specific sales data to allow weighting of the individual HSR values for each product within a category, but this type of data is expensive and was not available for this project.

The analysis was limited to only the 11 largest food and beverage manufacturers in India and by the availability of nutrient data. Not all companies elected to verify the nutrient data provided to them. While most companies complied with Indian labeling requirements, available data were mostly insufficient to apply the selected nutrient profile models given that certain nutrients (e.g., sodium) are not required to be labelled in India [17]. This resulted in imputation for some nutrient values which, because it was done at a FoodSwitch category level, is likely to have resulted in underestimation of the real differences between companies that produced similar types of food and beverages (for example, dairy). The imputation strategy we employed was a pragmatic compromise between enabling the inclusion of the majority of identified products versus basing analysis predominantly on proxy data. Due to differences in the nutrients required for calculation for each model, some products were eligible under one but not the other. The nutrient profile models selected take no account of serving size because there are no agreed international standards, but inclusion of this information may have enabled a fuller picture of the true differences between the levels of adverse nutrients each manufacturer contributed to the Indian food supply.

Products that are not required to carry a nutrition label were excluded because such products typically contribute little to nutrient intake. For some companies these products made up a significant proportion of sales value (i.e., 75% of Company D sales are plain tea).

Box 1 **Policy Recommendations.**

**Companies should:**

- Direct investment toward improving the healthiness of products by enhancing the product mix and reformulating unhealthy products to healthier compositions.
- Redirect marketing towards healthier products to assist in increasing the proportion of sales derived from these foods.
- Ensure labels comply with international standards i.e., Codex Alimentarius General Standard for the Labelling of Packaged Foods.

**The Government of India should:**

- Compile and maintain a national food composition database, allowing action areas to be identified, addressed, and progress monitored.
- Establish a government led program to reduce salt, sugar and harmful fats in the food supply.
- Develop and implement effective and enforceable legislation to prevent the marketing of unhealthy products to children [18].
- Extend nutrition labeling requirements to comply with minimum international standards. Recent addition of saturated and trans fat are positive, but sodium should also be required.
- Given growing evidence of harm and increased consumer interest globally, added sugar labeling may also be considered in any legislative reform.

## 6. Conclusions

While imperfect, these analyses almost certainly quantified real differences between the nutritional value of the product portfolios of leading food and beverage manufacturers in India. The findings are in broad alignment with prior perceptions about the relative healthiness of the product portfolios and are likely to reflect real differences. These data can helpfully contribute to comparative assessments of corporations such as that done by the Access To Nutrition Foundation, and may also guide future actions by companies and the Indian Government to support healthier diets (Box 1).

## Supplementary Materials

The following are available online at www.mdpi.com/2072-6643/9/10/1103/s1, Figure S1: Ranking of companies by (a) mean Health Star Rating (HSR); (b) proportion of healthy products (HSR ≥ 3.5) and (c) proportion of products eligible for marketing to children (all without sales-value-weighting); Table S1: Product categorization—Euromonitor sub-categories and equivalent HSR and WHO Euro categories by category number and description *; Table S2: Alignment of nutrients required for profiling models with those required by Indian labelling legislation; Table S3: Number of food and beverage products by company in Euromonitor sub-categories; Table S4: Distribution of Health Star Rating (HSR) by company; Table S5: Mean and range Health Star Rating (HSR) of products by Euromonitor sub-category, all manufacturers.
