# Supplementary material for: An Evaluation of the Healthiness of the Indian Packaged Food and Beverage Supply
Source: Nutrients. 2017 Oct 9;9(10):1103. doi: 10.3390/nu9101103 (PMC5691719; doi:10.3390/nu9101103)
Supplement: Supplementary file 1 [file nutrients-09-01103-s001.zip › nutrients-218120-supplementary.pdf]

nutrients

MDPI

Supplementary Figure S1. Ranking of companies by a) mean Health Star Rating (HSR), b) proportion of healthy products (HSR≥3.5) and c) proportion of products eligible for marketing to children (all without sales-value-weighting).

a) mean HSR

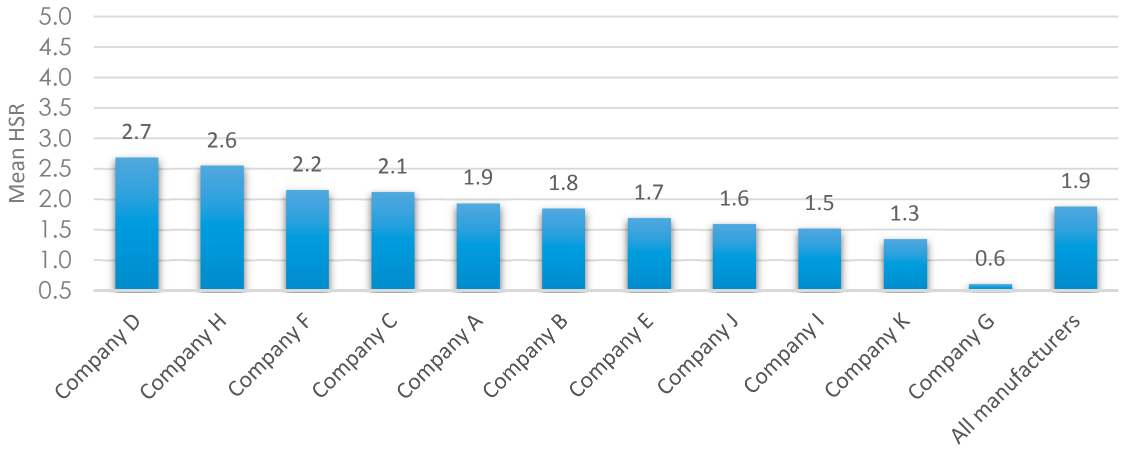

b) proportion of healthy products (HSR≥3.5)

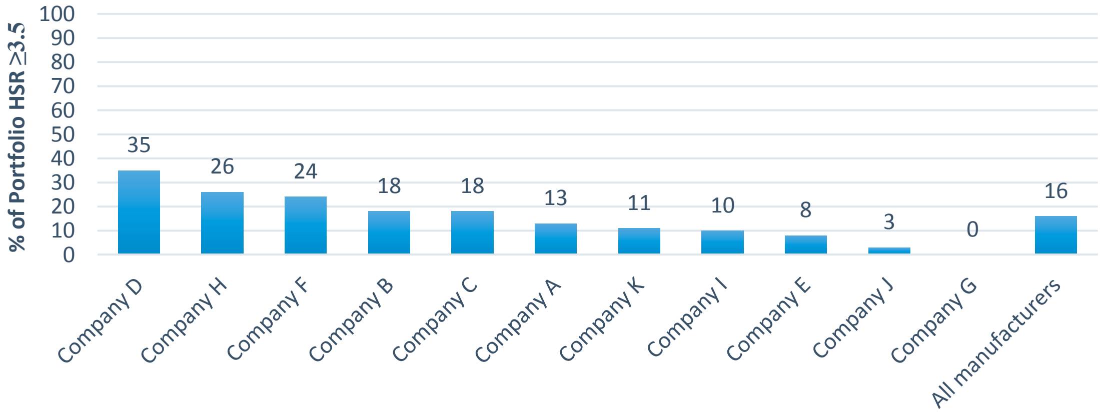

c) proportion of products eligible for marketing to children

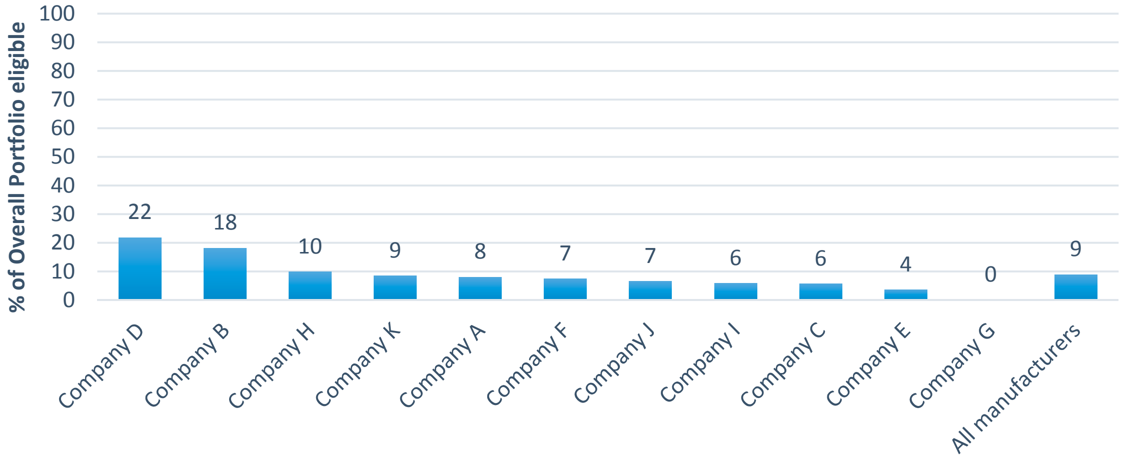

Supplementary Table S1. Product categorization—Euromonitor sub-categories and equivalent HSR and WHO Euro categories by category number and description*.

Numbers accompanying categories correspond to numeric codes assigned within each model's published guidance materials [9, 12].

Euromonitor Sub-category	HSR Category	WHO Euro category
Bread	2 (Non-Dairy foods)	11 (Bread, bread products and crisp breads)
Cake	2 (Non-Dairy foods)	2 (Cakes, sweet biscuits and pastries; other sweet bakery wares, and dry mixes for making such)
Savory biscuits	2 (Non-Dairy foods)	3 (Savory snacks)
Sweet biscuits	2 (Non-Dairy foods)	2 (Cakes, sweet biscuits and pastries; other sweet bakery wares, and dry mixes for making such)
Snack bars	2 (Non-Dairy foods)	1 (Chocolate and sugar confectionery, energy bars, and sweet toppings and desserts)
Breakfast cereals	2 (Non-Dairy foods)	6 (Breakfast cereals)
Confectionery	2 (Non-Dairy foods)	1 (Chocolate and sugar confectionery, energy bars, and sweet toppings and desserts)
Cheese	3D (Cheese)**<br>2 (Non-Dairy foods)	8 (Cheese)
Ice cream and frozen desserts	2 (Non-Dairy foods)***	5 (Edible ices)
Fats and oils	3 (Oils and spreads)	10 (Butter and other fats and oils)
Processed fruit and veg	2 (Non-Dairy foods)	16 (Processed fruit, vegetables and legumes)
Ready meals	2 (Non-Dairy foods)	9 (Ready-made and convenience foods and composite dishes)
Rice, pasta and noodles	2 (Non-Dairy foods)	12 (Fresh or dried pasta, rice and grains)
Cooking sauces	2 (Non-Dairy foods)	17 (Sauces, dips and dressings)
Table sauces	2 (Non-Dairy foods)	17 (Sauces, dips and dressings)
Soup	2 (Non-Dairy foods)	9 (Ready-made and convenience foods and composite dishes)
Spreads	2 (Non-Dairy foods)	Nut spreads: 1 (Chocolate and sugar confectionery, energy bars, and sweet toppings and desserts)<br>Jams: 16 (Processed fruit, vegetables and legumes)
Savory snacks	2 (Non-Dairy foods)	3 (Savory snacks)
Drinking milk products	1D (Dairy Beverages)	4b (Milk drinks)
Condensed milk	1D (Dairy Beverages)	4b (Milk drinks)
Dairy Whiteners	1D (Dairy Beverages)	4b (Milk drinks)
Yoghurt and sour milk	2D (Dairy Foods)	7 (Yoghurts, sour milk, cream and other similar foods)
Hot beverages	1 (Non-Dairy Beverage)	N/A
Regular cola carbonates	1 (Non-Dairy Beverage)	4d (Other beverages)
Low calorie cola carbonates	1 (Non-Dairy Beverage)	4d (Other beverages)
Lemonade/lime carbonates	1 (Non-Dairy Beverage)	4d (Other beverages)

Orange carbonates	1 (Non-Dairy Beverage)	4d (Other beverages)
Mixers	1 (Non-Dairy Beverage)	4d (Other beverages)
Other non-cola carbonates	1 (Non-Dairy Beverage)	4d (Other beverages)
Nectars	1 (Non-Dairy Beverage)	4a (Juices)
Juice drinks	1 (Non-Dairy Beverage)	4a (Juices)
Flavored bottled water	1 (Non-Dairy Beverage)	4d (Other beverages)
Sports drinks	1 (Non-Dairy Beverage)	4d (Other beverages)
Energy drinks	1 (Non-Dairy Beverage)	4c (Energy drinks)
Liquid concentrates	1 (Non-Dairy Beverage)	4d (Other beverages)
Powder concentrates	1 (Non-Dairy Beverage)	4d (Other beverages)
Ready-to-drink (RTD) tea	1 (Non-Dairy Beverage)	4d (Other beverages)
Fruit-flavored drinks	1 (Non-Dairy Beverage)	4d (Other beverages)

* For detailed definitions see: Euromonitor sub-categories http://www.euromonitor.com/about-us; HSR categories, HSR Guide for Industry (link below); WHO Euro categories, p 5-6 of guidance materials at http://www.euro.who.int/__data/assets/pdf_file/0005/270716/Europe-nutrient-profile-model-2015-en.pdf?ua=1

** Defined for the purposes of HSR as cheeses with calcium content ≥320mg/100g. Where insufficient calcium content, product classified in Category 2 foods.

*** Custards, desserts, cream cheese, ice-cream and cream are not considered as dairy foods but are classified as Category 2 foods for the purpose of HSR. For further explanation see the HSR Guide for Industry http://healthstarrating.gov.au/internet/healthstarrating/publishing.nsf/Content/guide-for-industry-document

Supplementary Table S2. Alignment of nutrients required for profiling models with those required by Indian labelling legislation.

	**Indian Regulations**	**HSR**	**WHO Euro**
Total number of nutrients required	5	8	5
Protein	✓	✓	
Fiber		✓	
Fruit and vegetable content		✓	
Energy	✓	✓	✓
Total fat	✓		✓
Saturated fat		✓	✓
Trans fat			✓
Carbohydrate	✓		
Total sugars	✓	✓	✓
Added sugars			✓
Other sweeteners			✓
Sodium		✓	✓
Calcium		✓	
Number of nutrients aligned to Indian legislation		3/5	3/5

Supplementary Table S3. Number of food and beverage products by company in Euromonitor sub-categories.

	EUROMONITOR SUBCATEGORY	Company A	Company B	Company C	Company D	Company E	Company F	Company G	Company H	Company I	Company J	Company K	Total
FOODS	Bread	7	0	0	0	0	0	0	0	0	0	0	**7**
	Cake	12	0	0	0	0	0	0	0	0	0	0	**12**
	Dessert mixes	0	0	0	0	2	0	0	0	0	0	0	**2**
	Savory biscuits	8	0	0	0	3	0	0	0	0	10	0	**21***
	Sweet biscuits	46	0	0	0	29	1	7	0	1	45	0	**128***
	Breakfast cereals	0	0	0	2	1	0	0	0	2	0	14	**19***
	Confectionery	0	0	3	0	4	0	29	0	13	12	0	**61***
	Cheese	7	0	9	0	0	1	0	4	0	0	0	**21***
	Drinking milk products	3	2	19	0	0	17	0	16	4	0	0	**62***
	Yoghurt and sour milk	3	0	5	0	0	3	0	14	8	0	0	**33***
	Ice cream and frozen desserts	0	0	41	34	0	27	0	56	0	0	0	**157***
	Fats and oils	1	0	5	0	1	4	0	7	0	0	0	**18***
	Processed fruit and vegetables	0	0	0	0	5	0	0	3	0	0	0	**8**
	Ready meals	0	0	1	0	17	0	0	1	3	0	0	**22**
	Rice, pasta and noodles	0	0	0	5	9	0	0	0	7	0	0	**21***
	Cooking sauces	0	0	0	17	9	0	0	0	6	0	0	**32***
	Table sauces	0	0	0	8	5	0	0	7	4	0	0	**24***
	Soup	0	0	0	19	0	0	0	0	0	0	0	**19**
	Spreads	0	0	0	7	1	0	0	4	0	0	0	**12**
	Savory snacks	0	0	0	0	19	0	0	0	0	9	92	**120***
	Other dairy products	0	0	1	0	0	0	0	2	1	0	0	4
	Whitener	0	1	2	0	0	1	0	1	3	0	0	8
	Condensed milk	0	0	1	0	0	0	0	0	2	0	0	3
BEVERAGES	Regular cola carbonates	0	2	0	0	0	0	0	0	0	0	1	**3**
	Low calorie cola carbonates	0	2	0	0	0	0	0	0	0	0	1	**3**
	Lemonade/lime carbonates	0	4	0	0	0	0	0	0	0	0	2	**6**
	Orange carbonates	0	1	0	0	0	0	0	0	0	0	1	**2**
	Mixers	0	5	0	0	0	0	0	0	0	0	2	**7**
	Other non-cola carbonates	0	3	0	0	0	0	0	0	0	0	1	**4**
	Nectars (25-99% juice)	0	1	0	6	3	0	0	0	0	0	0	**10**
	Low calorie cola carbonates	0	2	0	0	0	0	0	0	0	0	1	**3**
	Juice drinks (up to 24% juice)	0	7	0	0	5	0	0	7	0	0	19	**38***
	Still bottled water	0	2	0	0	0	0	0	0	0	0	1	**3**
	Sports drinks	0	0	0	0	0	0	0	0	0	0	3	**3**
	Liquid concentrates	0	0	0	1	0	0	0	0	2	0	0	**3**
	Powder concentrates	0	0	0	2	0	0	7	0	4	0	2	**15**
	Ready-to-drink tea	0	2	0	1	0	0	0	0	0	0	2	**5**
	Hot beverages	0	1	1	8	0	0	5	0	12	0	0	**27***
SUBTOTAL FOOD		**87**	**3**	**87**	**92**	**105**	**54**	**36**	**115**	**53**	**76**	**106**	**814**
SUBTOTAL BEVERAGE		**0**	**30**	**1**	**18**	**8**	**0**	**12**	**7**	**18**	**0**	**35**	**129**
TOTAL FOOD AND BEVERAGE		**87**	**33**	**88**	**110**	**113**	**54**	**48**	**122**	**71**	**78**	**141**	**943**

*Shaded subcategories mark those with sufficient products (at least five products made by at least two companies) for sub-category analysis.

Supplementary Table S4. Distribution of Health Star Rating (HSR) by company.

	Health Star Rating										
	0.5	1	1.5	2	2.5	3	3.5	4	4.5	5	Total
Company A	11	8	23	23	2	9	4	6	1	0	87
Company B	6	11	5	4	0	1	1	0	0	5	33
Company C	12	10	8	19	15	6	5	8	1	1	85
Company D	4	12	8	12	7	24	13	23	0	0	103
Company E	17	23	26	17	13	5	4	5	0	0	110
Company F	8	8	11	3	4	4	0	7	4	1	50
Company G	41	2	2	0	1	0	0	0	0	0	46
Company H	4	7	23	27	20	8	7	8	12	4	120
Company I	35	3	5	5	4	9	1	4	2	0	68
Company J	14	12	26	5	8	8	1	1	0	0	75
Company K	70	20	20	8	4	4	1	3	7	4	141
Total no. of products	222	116	157	123	78	78	37	65	27	15	918
% of total products	24.2%	12.5%	17.1%	13.4%	8.5%	8.5%	4.0%	7.1%	2.9%	1.8%	100%

Supplementary Table S5. Mean and range Health Star Rating (HSR) of products by Euromonitor sub-category, all manufacturers

	Euromonitor Subset	Mean HSR	Range HSR
FOODS	**Bread**	3.8	**3.5 to 4.0**
	**Cake**	1.4	**0.5 to 2.0**
	**Dessert mixes**	2.3	**2.0 to 2.5**
	**Savory biscuits**	2.0	**1.5 to 3.0**
	**Sweet biscuits**	1.5	**0.5 to 4.0**
	**Breakfast cereals**	3.1	**0.5 to 5.0**
	**Confectionery**	0.7	**0.5 to 3.0**
	**Cheese**	2.8	**0.5 to 5.0**
	**Drinking milk products**	2.8	**0.5 to 5.0**
	**Yoghurt and sour milk**	3.5	**2.0 to 4.5**
	**Ice cream and frozen desserts**	2.0	**0.5 to 4.0**
	**Fats and oils**	1.2	**0.5 to 4.5**
	**Processed fruit and vegetables**	4.0	**2.5 to 5.0**
	**Ready meals**	1.5	**1.0 to 4.0**
	**Rice, pasta and noodles**	2.5	**0.5 to 4.0**
	**Cooking sauces**	2.5	**0.5 to 4.5**
	**Table sauces**	2.8	**1.5 to 4.5**
	**Soup**	3.8	**0.5 to 4.0**
	**Spreads**	2.0	**1.5 to 3.5**
	**Savory snacks**	1.3	**0.5 to 4.5**
	**Other dairy**	1.3	**0.5 to 1.5**
	**Whitener**	1.2	**0.5 to 3.5**
	**Condensed milk**	1.3	**0.5 to 3.0**
BEVERAGES	**Regular cola carbonates**	1.0	**1.0**
	**Low calorie cola carbonates**	2.0	**2.0**
	**Lemonade/lime carbonates**	1.2	**1.0 to 2.0**
	**Orange carbonates**	0.8	**0.5 to 1.0**
	**Mixers**	4.0	**1.0 to 5.0**
	**Other non-cola carbonates**	0.9	**0.5 to 1.0**
	**Nectars (25-99% juice)**	1.0	**0.5 to 2.0**
	**Juice drinks (up to 24% juice)**	0.9	**0.5 to 2.0**
	**Still bottled water**	5.0	**5.0**
	**Sports drinks**	1.5	**1.5**
	**Liquid concentrates**	0.5	**0.5**
	**Powder concentrates**	0.7	**0.5 to 1.5**
	**Ready-to-drink tea**	1.4	**1.0 to 2.0**
	**Hot beverages**	0.7	**0.5 to 4.0**
